# Association of Different Risk Scores and 30-Day Mortality in Kidney Transplant Recipients with COVID-19

**DOI:** 10.3390/medicina59040657

**Published:** 2023-03-26

**Authors:** Josipa Domjanović, Tea Domjanović Škopinić, Andrija Matetic

**Affiliations:** 1 Department of Nephrology, University Hospital of Split, 21000 Split, Croatia; 2 Department of Cardiology, University Hospital of Split, 21000 Split, Croatia; 3 School of Medicine, University of Split, 21000 Split, Croatia

**Keywords:** kidney transplant recipients, COVID-19, risk score, mortality

## Abstract

*Background and Objectives*: Clinical risk scores were poorly examined in kidney transplant recipients (KTR) with COVID-19. *Materials and Methods*: This observational study compared the association and discrimination of clinical risk scores (MEWS, qCSI, VACO, PSI/PORT, CCI, MuLBSTA, ISTH-DIC, COVID-GRAM and 4C) with 30-day mortality in 65 hospitalized KTRs with COVID-19. Cox regression was used to derive hazard ratios (HR) and 95% confidence intervals (95% CI), and discrimination was assessed by Harrell’s C. *Results*: A significant association with 30-day mortality was demonstrated for MEWS (HR 1.65 95% CI 1.21–2.25, *p* = 0.002); qCSI (HR 1.32 95% CI 1.15–1.52, *p* < 0.001); PSI/PORT (HR 1.04 95% CI 1.02–1.07, *p* = 0.001); CCI (HR 1.79 95% CI 1.13–2.83, *p* = 0.013); MuLBSTA (HR 1.31 95% CI 1.05–1.64, *p* = 0.017); COVID-GRAM (HR 1.03 95% CI 1.01–1.06, *p* = 0.004); and 4C (HR 1.79 95% CI 1.40–2.31, *p* < 0.001). After multivariable adjustment, significant association persisted for qCSI (HR 1.33 95% CI 1.11–1.59, *p* = 0.002); PSI/PORT (HR 1.04 95% CI 1.01–1.07, *p* = 0.012); MuLBSTA (HR 1.36 95% CI 1.01–1.85, *p* = 0.046); and 4C Mortality Score (HR 1.93 95% CI 1.45–2.57, *p* < 0.001) risk scores. The best discrimination was observed with the 4C score (Harrell’s C = 0.914). *Conclusions*: Risk scores such as qCSI, PSI/PORT and 4C showed the best association with 30-day mortality amongst KTRs with COVID-19.

## 1. Introduction

Novel coronavirus disease (COVID-19) produced a global health burden, leading to high morbidity and mortality [1]. Specific patient populations particularly suffered from COVID-19, such as those with chronic kidney disease (CKD) and kidney transplant recipients (KTR) [2,3].

KTRs conveyed a notably elevated risk of severe COVID-19 with a mortality rate ranging from 18.6% to 44.3% [4,5,6]. Adverse outcomes of COVID-19 in KTRs could be attributed to different mechanisms. Chronic immunosuppressive therapy and susceptibility to infections in this fragile population represent a substantial challenge to the healthcare system [4,7,8,9]. Furthermore, the modification of immunosuppressive therapy, which is often carried out during COVID-19, was previously associated with acute graft failure [10,11]. On the other hand, chronic immunosuppressive therapy could presumably modulate immunologic response in COVID-19 and prevent cytokine storm, which was previously associated with renal injury [7,10,11,12]. It has, therefore, been suggested that KTRs have similar outcomes to the general population with COVID-19 when adjusted for baseline differences [13,14].

Bearing in mind the heterogeneity of the KTR population, proper risk stratification represents an important decision-making step during COVID-19 management. Easy-to-use risk scores could improve the detection of patients at risk of short-term adverse outcomes and advocate closer follow-up. Numerous risk scores were previously examined in a general COVID-19 population, but KTR patients lack validation of such useful risk stratification tools. Particularly valuable instruments account for different patient factors such as demographic characteristics and comorbidities, together with clinical, radiologic and laboratory parameters [15,16].

Therefore, this study aimed to compare the performance of several risk scores for 30-day post-discharge mortality in the KTR population, including the *Modified early warning score* (MEWS); *Quick COVID-19 Severity Index* (qCSI); *Veterans Health Administration COVID-19* (VACO) score; *Pneumonia Severity Index for community-acquired pneumonia/Pneumonia Patient Outcomes Research Team cohort study* (PSI/PORT) score; Charlson Comorbidity Index (CCI); *Multilobular infiltration*, *hypo-Lymphocytosis, Bacterial coinfection*, *Smoking history*, *hyper-Tension and Age* (MuLBSTA) score; *International Society on Thrombosis and Haemostasis Criteria for Disseminated Intravascular Coagulation* (ISTH-DIC) score; *COVID-GRAM Critical Illness Risk* score (COVID-GRAM); and *Coronavirus Clinical Characterisation Consortium Mortality* score (4C Mortality).

## 2. Materials and Methods

### 2.1. Ethical and Institutional Considerations

The study protocol was approved by the *Medical Research Ethical Committee*. The clinical research was performed by the ethical standards of the *Declaration of Helsinki*.

### 2.2. Study Design and Patients

This single-centre observational retrospective study included 65 adult KTRs of both sexes who were treated for COVID-19 at the *University Hospital* in the period from August 2020 to March 2022. All patients were tested using the reverse transcription-polymerase chain reaction (RT-PCR) test (Liferiver Novel Coronavirus RT-PCR Kit/MIC qPCR cycler) with the specific 3-sequence (E-gen; ORF 1ab-gen; N-gen). Patients with incomplete medical documentation and those who continued treatment in other institutions and were consequently lost to follow-up were excluded (N = 2). The flow diagram of the study is graphically presented (Supplementary Figure S1). The study was reported according to the *Strengthening the Reporting of Observational Studies in Epidemiology* (STROBE) guidelines (Appendix A).

### 2.3. Data Source and Patient Assessment

Using the electronic medical records, baseline characteristics (age, sex, body weight, body height); clinical parameters at the time of admission (systolic blood pressure (SBP), heart rate (HR), respiratory rate (RR), level of consciousness, oxygen saturation, body temperature, comorbidities, smoking history, medications and transplant duration); laboratory parameters (white blood cells (WBC), platelets, red blood cells (RBC), hematocrit, D-dimer, fibrinogen, lactate dehydrogenase (LDH), direct bilirubin, blood urea nitrogen (BUN), C-reactive protein (CRP), sodium, glucose); radiologic findings; and COVID-19-related data (duration of symptoms, specific anti-inflammatory and antiviral therapy, chronic immunosuppressive therapy, vaccination status) were collected. Acute kidney injury during follow-up was defined as an increase in serum creatinine by ≥26.5 umol/L, or an increase in serum creatinine ≥1.5 times compared to baseline values.

### 2.4. Risk Scores of Interests

All risk scores were calculated as recommended in the derivation and validation studies, using the appropriate patient-level data [15,16,17,18,19,20,21,22,23,24,25,26,27,28,29,30,31]. Elementary data about risk scores are shown in Supplementary Table S1. The data for risk score assessment were determined using the data from the point of hospital admission.

The MEWS risk score was developed in 2001 by Subbe et al. to detect critically ill patients upon admission to the emergency department [17]. It also proved to be an effective tool in predicting 28-day mortality in the general population of COVID-19 hospitalized patients [18]. It is fully based on clinical parameters (SBP, HR, RR, temperature, AVPU (alert, verbal, pain, unresponsive) score). Risk score ranges from 0 to 14, while a score >5 was associated with increased mortality within 60 days of admission to an intensive care unit (ICU) [17].

The qCSI risk score was developed by Haimovich et al. for the prediction of respiratory failure within 24 h after hospital admission. This score is composed of respiratory parameters (RR, oxygen saturation and oxygen flow rate). The risk score ranges from 0 to 12, and the original study showed that a risk score >9 was associated with up to 57% increased risk of disease progression [19]. qCSI showed good performance in predicting hospital mortality in the general COVID-19 patient population [20].

The CCI risk score is one of the oldest comorbidity risk scores, initially developed in 1987, for measuring the comorbidity burden that is associated with patient mortality [21]. It is composed of age and various comorbidities (myocardial infarction, chronic heart failure, peripheral vascular disease, dementia, chronic pulmonary disease, connective tissue disease, liver disease, peptic ulcer disease, diabetes mellitus, chronic kidney disease, cancer history, acquired immunodeficiency syndrome (AIDS) and hematologic disease). It ranges from 0 to 37 points, while the final score is expressed as a percentage of estimated 10-year survival [21]. Authors Kuswardhani et al. suggested that CCI should be used as a risk stratification tool in COVID-19 hospitalized patients [22].

VACO is a combined risk score encompassing demographic characteristics (age, sex) and individual components of CCI. The original study showed good performance for the prediction of fatal events in COVID-19 [15].

The PSI/PORT risk score was initially derived for the prediction of 30-day in-hospital mortality and was thereafter validated in the *Pneumonia PORT cohort study* [23]. It could detect patients with a low risk of pneumonia-associated mortality and assess the need for hospitalization in this patient population. This score divides patients into 5 risk groups based on the 30-day mortality rate. It is based on demographic characteristics (age, sex, residency in nursing home); comorbidities (cancer history, liver disease, chronic heart failure, cerebrovascular disease, renal disease); clinical parameters (RR, SBP, body temperature, HR); laboratory parameters (BUN, sodium, glucose, hematocrit, oxygen partial pressure); and radiologic findings (pleural effusion) [23].

The MuLBSTA risk score was recently derived as a simple tool for the prediction of viral pneumonia-associated 90-day mortality. It is composed of radiologic findings, lymphocyte count, bacterial co-infection, smoking history, age and history of arterial hypertension. The risk score ranges from 0 to 20 [24]. Several studies have reported satisfactory detection of high-risk patients who are at risk of developing severe forms of COVID-19 [25,26].

ISTH-DIC was developed in 2001 to improve disseminated intravascular coagulation (DIC) management but was subsequently examined in different clinical settings. The risk score ranges from 0 to 8 and scores ≥5 are associated with a high probability of DIC [27]. In the setting of the COVID-19, Anwar et al. tested this risk score for the prediction of mortality in COVID-19 patients [28].

The COVID-GRAM risk score was developed to detect patients that will require intensive care unit admission, mechanical ventilation or fatal outcome. This score includes radiologic findings, age, specific symptoms, comorbidities and laboratory parameters (neutrophil-to-lymphocyte ratio, LDH, direct bilirubin) [29].

The 4C Mortality Score was developed for the prediction of mortality in hospitalized COVID-19 patients. It was derived from a prospective study on 57,824 patients and includes demographic characteristics, comorbidities, clinical parameters and laboratory findings. The risk score ranges from 0 to 20 and divides patients into 4 risk groups based on the in-hospital mortality [16]. Several external validation studies showed satisfactory performance of this risk score in assessing mortality risk [30,31].

### 2.5. Outcomes

A primary outcome was 30-day post-discharge all-cause mortality (fatal events) and its association with different risk scores (MEWS, qCSI, VACO, PSI/PORT, CCI, MuLBSTA, ISTH-DIC, COVID-GRAM, 4C Mortality Score). Other outcomes included calibration and discrimination of the risk scores with regard to 30-day post-discharge all-cause mortality.

### 2.6. Statistical Analysis

Continuous data were presented as median (interquartile range (IQR)), while categorical variables were expressed as numbers (percentages). The Cox proportional hazards regression analysis was used (univariate model) to determine the association of a selected risk score with 30-day post-discharge mortality during the study period. The results were expressed as hazard ratios (HR) and 95% confidence intervals (95% CI), which correspond to a 1-unit change of each score on a continuous scale. An additional multivariable Cox proportional hazards regression analysis was performed to adjust for potential confounders for each score. Due to differences across risk scores and to prevent multicollinearity, each multivariable model was specifically designed for each risk score. The models were adjusted for the following variables: MEWS (SpO_2_, CRP, chronic heart failure); qCSI (HR, SBP, CRP, chronic heart failure); VACO (HR, SBP, SpO_2_, CRP); PSI/PORT (CRP); CCI (HR, SBP, SpO_2_, CRP); MuLBSTA (HR, SBP, SpO_2_, CRP, chronic heart failure); ISTH-DIC (HR, SBP, SpO_2_, CRP, chronic heart failure); COVID-GRAM (HR, SBP, SpO_2_, CRP); and 4C (HR, SBP). Furthermore, as a general measure of the predictive accuracy of selected risk scores, the receiver operating characteristic (ROC) and area under the curve (AUC) were calculated. Youden’s index was then calculated to determine the specific cut-off point with optimal sensitivity/specificity ratio for each risk score. The difference between areas was determined using a method by Hanley and McNeil [32]. Calibration/goodness-of-fit was assessed by the Hosmer–Lemeshow test and visually by calibration plots, while the discrimination was assessed by Harrell’s C concordance and Somers’ D index. A two-sided *p*-value of <0.05 was considered significant. The statistical data analysis was carried out using a Statistical Package for the Social Sciences (SPSS) software (IBM Corp, New York, NY, USA; version 20) and Stata software (StataCorp, College Station, TX, USA; version 17).

## 3. Results

The study cohort mostly consisted of middle-aged (57 years) female patients (66.2%) with a transplant duration of 8 years (Table 1). Most enrolled patients (70.8%) had X-ray-confirmed COVID-19-associated pneumonia (Supplementary Table S2).

**Figure 1:**
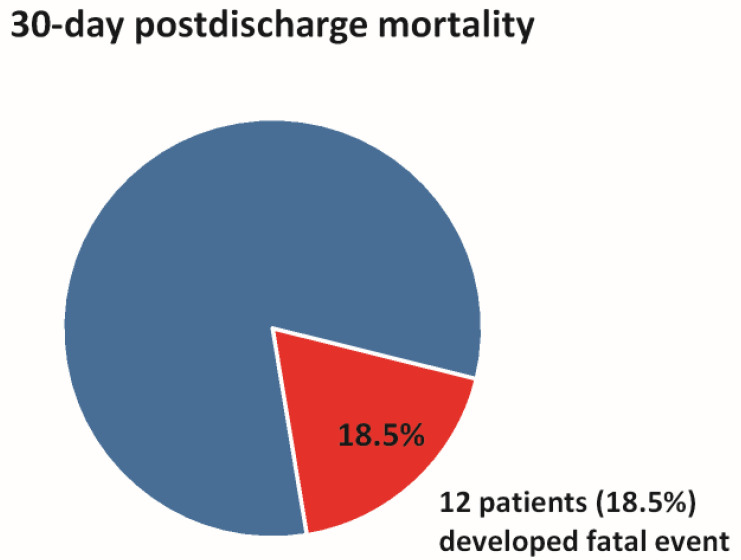
Overview of the 30-day post-discharge mortality in the study sample.

A total of 12 patients (18.5%) developed 30-day all-cause mortality (Figure 1). When comparing baseline characteristics between survival and mortality group, there was no statistically significant between-group difference, except in heart rate, systolic blood pressure, oxygen saturation, CRP and presence of chronic heart failure (*p* < 0.05). Importantly, the mortality group exhibited a significantly higher occurrence of acute kidney injury (N = 2, 3.8% vs. N = 6, 50.0%, *p* < 0.001) (Table 2).

Out of the examined risk scores, a significant association with 30-day post-discharge mortality was demonstrated for MEWS (HR 1.65 95% CI 1.21–2.25, *p* = 0.002); qCSI (HR 1.32 95% CI 1.15–1.52, *p* < 0.001); PSI/PORT (HR 1.04 95% CI 1.02–1.07, *p* = 0.001); CCI (HR 1.79 95% CI 1.13–2.83, *p* = 0.013); MuLBSTA (HR 1.31 95% CI 1.05–1.64, *p* = 0.017); COVID-GRAM (HR 1.03 95% CI 1.01–1.06, *p* = 0.004); and 4C Mortality Score (HR 1.79 95% CI 1.40–2.31, *p* < 0.001) risk scores (Table 3).

After multivariable adjustment for the differing baseline characteristics between the survival and the mortality group, significant association with 30-day post-discharge mortality was consistent for the qCSI (HR 1.33 95% CI 1.11–1.59, *p* = 0.002); PSI/PORT (HR 1.04 95% CI 1.01–1.07, *p* = 0.012); MuLBSTA (HR 1.36 95% CI 1.01–1.85, *p* = 0.046); and 4C Mortality Score (HR 1.93 95% CI 1.45–2.57, *p* < 0.001) risk scores (Table 4).

**Figure 2:**
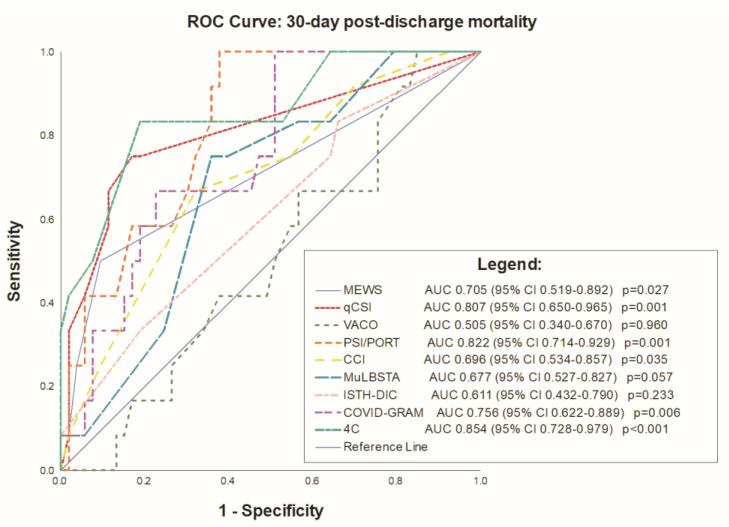
Receiver operating characteristics of the risk scores. **Abbreviations**: 4C Mortality Score—Coronavirus Clinical Characterisation Consortium Mortality Score; AUC—Area Under the Curve; CCI—Charlson Comorbidity Index; COVID-GRAM—COVID-19 Critical Illness Prediction; ISTH-DIC—The International Society on Thrombosis and Haemostasis-Disseminated Intravascular Coagulation; MEWS—The Modified Early Warning Score; MuLBSTA—Multilobular infiltration, hypo-Lymphocytosis, Bacterial coinfection, Smoking history, hyper-Tension and Age; OR—odds ratio; PSI/PORT—The Pneumonia Severity Index; qCSI—Quick COVID-19 Severity Index; ROC—Receiver Operator Characteristics; VACO—Veterans Health Administration COVID-19 Index.

A significant predictive accuracy was consistently shown for the MEWS (AUC 0.705 95% CI 0.519–0.892, *p* = 0.027); qCSI (AUC 0.807 95% CI 0.650–0.965, *p* = 0.001); PSI/PORT (AUC 0.822 95% CI 0.714–0.929, *p* = 0.001); CCI (AUC 0.696 95% CI 0.534–0.857, *p* = 0.035); COVID-GRAM (AUC 0.756 95% CI 0.622–0.889, *p* = 0.006); and 4C Mortality Score (AUC 0.854 95% CI 0.728–0.979, *p* < 0.001) risk scores (Figure 2 and Table 5).

**Figure 3:**
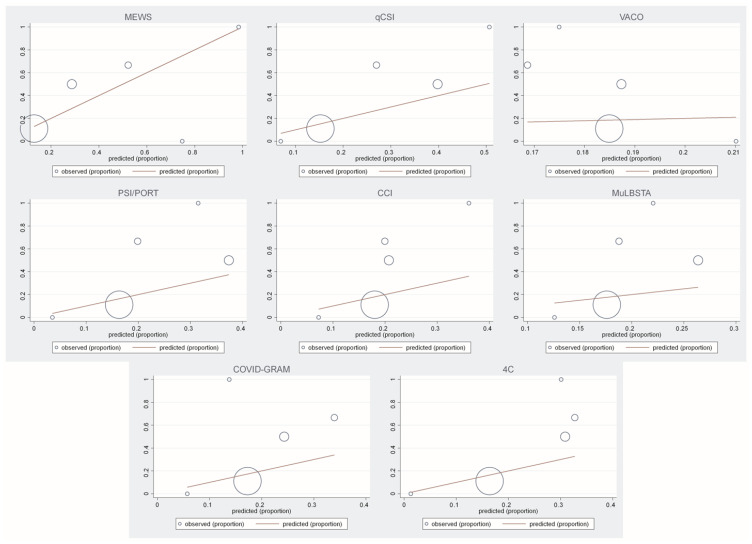
Calibration plots. **Abbreviations**: 4C Mortality Score—Coronavirus Clinical Characterisation Consortium Mortality Score; CCI—Charlson Comorbidity Index; COVID-GRAM—COVID-19 Critical Illness Prediction; ISTH-DIC—The International Society on Thrombosis and Haemostasis-Disseminated Intravascular Coagulation; MEWS—The Modified Early Warning Score; MuLBSTA—Multilobular infiltration, hypo-Lymphocytosis, Bacterial coinfection, Smoking history, hyper-Tension and Age; OR—odds ratio; PSI/PORT—The Pneumonia Severity Index; qCSI—Quick COVID-19 Severity Index; ROC—Receiver Operator Characteristics; VACO—Veterans Health Administration COVID-19 Index.

All risk scores demonstrated satisfactory calibration with regards to 30-day post-discharge mortality, except COVID-GRAM (Supplementary Table S3 and Figure 3). When comparing the differences between the areas of the risk scores, a significant distinction was observed only between 4C Mortality Score and CCI (0.158, *p* = 0.010), while there was no difference in the other risk scores (Supplementary Table S4).

A satisfactory discrimination of the risk scores with regards to 30-day post-discharge mortality was confirmed for the MEWS (Harrell’s C 0.694); qCSI (Harrell’s C 0.809); PSI/PORT (Harrell’s C 0.833); CCI (Harrell’s C 0.785); MuLBSTA (Harrell’s C 0.678); and COVID-GRAM (Harrell’s C 0.748) risk scores, while the best overall discrimination was observed with the 4C Mortality Score (Harrell’s C 0.914 and Somers’ D 0.829) (Table 3).

## 4. Discussion

To the best of our knowledge, this is the first study to compare the performance of these risk scores for mortality amongst the KTR population hospitalized for COVID-19. Risk stratification of this heterogenous patient population is valuable, warranting risk scores that could improve physicians’ recognition of patients at risk of adverse outcomes. This study reported several main findings. First, the 30-day all-cause mortality of KTRs with COVID-19 was substantially high, up to 18.5%. Second, several risk scores such as MEWS, qCSI, PSI/PORT, CCI, MuLBSTA, COVID-GRAM and 4C Mortality Score showed significant association with mortality. Third, the ROC curve analysis revealed a significant predictive accuracy for 30-day all-cause mortality with all risk scores, except MuLBSTA. Finally, the best overall calibration regarding 30-day post-discharge mortality was observed with the 4C Mortality Score.

The available literature is lacking validated risk stratification tools amongst the KTR population with COVID-19, which could be valuable for the detection of patients at risk of worse outcomes [4,7,8,9]. Most of the available risk scores were derived and validated in the general population and were not evaluated in this patient subgroup. Risk scores developed for the risk stratification of COVID-19 patients in the general population such as qCSI, COVID-GRAM and 4C Mortality Score also showed good performance in this fragile patient population. Interestingly, risk scores derived from the general population such as MEWS, PSI/PORT and CCI also showed a good association with 30-day post-discharge mortality in COVID-19 KTR patients. Similar results were reported when evaluating these risk scores in the general COVID-19 population [18,22,25,26].

Almost all studied risk scores were based on demographic characteristics and comorbidities, while only a few of them accounted for clinical, radiologic and laboratory parameters. Importantly, this study revealed the best performance with qCSI risk score, which is based exclusively on clinical parameters, as well as 4C Mortality Score, which additionally includes demographic factors, laboratory parameters and comorbidities.

The VACO risk score did not show satisfactory performance for mortality in KTR, although it was developed in the general COVID-19 population. It was developed in the early phases of the COVID-19 pandemic, which could have led to an overestimation of mortality risk that was observed in later validation studies [15,33]. The current study showed consistent findings of an inadequate performance of the VACO score in the COVID-19 KTR population. However, bearing in mind that VACO also includes age and CCI score, which were per se associated with mortality, these findings were unexpected [34,35]. A recent meta-analysis by Udomkarnjananun et al. showed that KTR non-survivors with COVID-19 were significantly older than their counterparts [36]. Nevertheless, further studies are needed to assess the utility of VACO risk score in the KTR population with COVID-19, particularly after the modification with different biomarkers such as procalcitonin or presepsin, which proved to improve the VACO index alone in predicting 30-day mortality risk in COVID-19 patients [37].

COVID-19 is a multisystem disease with a high affinity for vascular endothelium, which may lead to endothelial dysfunction and DIC [38,39,40]. Therefore, a risk score such as ISTH-DIC was suggested as a potential instrument in COVID-19. Nevertheless, it did not show satisfactory performance amongst the KTR population in this study. Consistent results in the overall COVID-19 population were previously reported by Anwar et al., which may be attributed to different pathophysiological mechanisms of COVID-19-associated DIC [28].

PSI/PORT previously yielded the best performance for in-hospital mortality amongst the general COVID-19 population compared to the qCSI, 4C Mortality Score and quick Sequential Organ Failure Assessment (SOFA) score [31]. This study consistently showed a significant association of PSI/PORT with 30-day post-discharge mortality, but the highest effect size was observed with the 4C Mortality Score.

There are a few clinical implications of this study. First, it could help improve the treatment of this patient population and enable the timely identification of KTR patients at risk of severe COVID-19. This could also enable closer follow-up of patients at risk. Second, it has hypothesis-generating purposes encouraging the further validation of risk scores in the KTR population, but also other special populations such as solid organ transplant recipients. Some of the available risk scores, such as the 4C Mortality Score, showed particularly promising features in the setting of COVID-19 and KTR. Third, this study could act as an incentive to derive a novel risk score, especially for this fragile patient population.

The potential application of risk scores in the KTR population shares some features with the general population with COVID-19, but it also has important specificities. The importance of clinical parameters (oxygen saturation, blood pressure, etc.) in patients with COVID-19 is universal and probably carries a similar prognostic role in both KTR and the general population. This is analogous to the comorbidity burden or intensity of laboratory deviations. Therefore, risk scores containing clinical parameters and comorbidities could offer similar utility in both patient groups. On the other hand, KTRs are, by definition, immunosuppressed chronically ill individuals that are more prone to homeostatic imbalance in acute stress states. Accordingly, combined risk scores that account for various patient-related factors (age, comorbidities, kidney function, etc.); COVID-19-related factors (intensity of inflammation, disease duration, co-infections, etc.); and transplant-related factors (transplant duration, medications, etc.) may offer more benefits for the KTR population.

Similar research is warranted to improve the outcomes of the KTR population. Previous large studies have shown that KTR exhibits impaired prognosis with COVID-19 [4,5,6]. This has been confirmed in more contemporary studies that showed a high in-hospital mortality (27–38%) [41,42]. This has been further reassured by findings that a fatal event usually occurs within 1 month of disease contraction [10]. While available data indicate that the in-hospital mortality of KTRs with COVID-19 did not changes substantially across pandemic phases, post-discharge mortality was increased during the first wave of COVID-19 pandemic compared to the second wave (24% vs. 16%) [41,43]. This emphasizes the need for proper risk management during the in-hospital period to improve the prognosis of these patients.

There are several limitations of this study. First, a relatively limited sample size from a single centre could affect the statistical strength of the study. The inherent limitations of single-centre studies, such as the lack of external validation and treatment bias, should be acknowledged. Second, the examined risk scores were mostly developed in the non-COVID-19 or general COVID-19 population, which raises the question of the need for external validation in specific settings such as the KTR population. Third, one should keep in mind a potential selection bias due to the retrospective design of the study. Finally, none of the evaluated risk scores included chronic immunosuppressive therapy, which could affect the disease course in this patient population.

## 5. Conclusions

In conclusion, risk scores such as qCSI, PSI/PORT and 4C Mortality Score showed the best association with 30-day post-discharge mortality amongst KTR patients with COVID-19, including satisfactory discrimination and calibration. Further powered multicentric longitudinal studies should reassess the utility and applicability of these risk scores in this fragile population.

## Figures and Tables

**Table 1:** Baseline characteristics of the study sample.

Variables	Study Sample (N = 65)
Age (years)	57 (45–66)
Female sex	43 (66.2%)
Systolic blood pressure (mmHg)	130 (126–140)
Diastolic blood pressure (mmHg)	78 (75–89)
Heart rate (/min)	80 (76–93)
SpO_2_ (%)	96 (95–96)
Transplant duration (years)	8 (2–11)
Single-dose Pfizer-BioNTech COVID-19 vaccination	3 (4.6%)
Double-dose Pfizer-BioNTech COVID-19 vaccination	11 (16.9%)
Symptom duration upon hospital admission (days)	7 (3–11)
Follow-up (days)	38 (35–42)
**Comorbidities**	
Arterial hypertension	53 (81.5%)
Diabetes mellitus	18 (27.7%)
Chronic heart failure	5 (7.7%)
Active smoking	5 (7.7%)
Atrial fibrillation	7 (10.8%)
Prior AMI	7 (10.8%)
Prior CVI	6 (9.2%)
PAD	10 (15.4%)
COPD/asthma	4 (6.2%)
**Laboratory parameters**	
WBC (×10^9^/L)	6.5 (5.3–8.9)
RBC (×10^12^/L)	4.7 (4.4–4.8)
Hgb (g/L)	128.5 (125.0–134.3)
Platelets (×10^3^/L)	240.1 (198.0–262.3)
CRP (mmol/L; maximal values)	72.6 (34.8–121.8)
BUN (mmol/L)	9.8 (8.0–15.3)
Creatinine (μmol/L)	128.5 (111.5–215.0)
eGFR (ml/min/1.73 m^2^)	44.5 (24.8–63.8)
D-dimers (mmol/L)	0.8 (0.6–1.7)
**Chronic immunosuppressive therapy**	
Mycophenolate Mofetil	58 (89.2%)
Azathioprine	1 (1.5%)
Cyclosporine	20 (30.8%)
Tacrolimus	36 (55.4%)
Everolimus	8 (12.3%)
Sirolimus	3 (4.6%)
Prednisone	65 (100.0%)
**COVID-19-related therapy**	
Reconvalescent plasma	6 (9.2%)
Casirivimab/Imdevimab	2 (3.1%)
Remdesivir	44 (67.7%)
Oxygen therapy	24 (36.9%)
**Transplant-related therapy**	
Mycophenolate + Tacrolimus + Prednison	34 (52.3%)
Mycophenolate + Cyclosporine + Prednison	18 (27.7%)
Mycophenolate + Everolimus + Prednison	4 (6.3%)
Mycophenolate + Sirolimus + Prednison	3 (4.6%)
Tacrolimus + Everolimus + Prednison	3 (4.6%)
Cyclosporine + Everolimus + Prednison	1 (1.5%)
Azathioprine + Prednison	1 (1.5%)
Cyclosporine + Prednison	1 (1.5%)
**Transplant-related therapy in the setting of COVID-19**	
Corticosteroid dose increase	65 (100.0%)
Calcineurin dose modification	
No dose modification	38 (58.5%)
Dose reduction	24 (36.9%)
Drug removal	3 (4.6%)
Antimetabolite dose modification	
No dose modification	6 (9.2%)
Dose reduction	24 (36.9%)
Drug removal	35 (53.8%)

Data are expressed as number (percent) or median (interquartile range). **Abbreviations**: AMI—acute myocardial infarction; BUN—blood urea nitrogen; COPD—chronic obstructive pulmonary disease; CRP—C-reactive peptide; CVI—cerebrovascular incident; eGFR—estimated glomerular filtration rate; Hgb—haemoglobin; LDH—lactate dehydrogenase; PAD—peripheral arterial disease; RBC—red blood cells; WBC—white blood cells.

**Table 2:** Baseline characteristics of the study sample.

Variables	Survival Group (N = 53)	Mortality Group (N = 12)	*p*-Value
Age (years)	61 (51–68)	73 (62–75)	0.063
Female sex	18 (34.0%)	4 (33.3%)	0.967
Systolic blood pressure (mmHg)	135 (130–150)	120 (117–126)	0.012
Diastolic blood pressure (mmHg)	80 (75–90)	73 (70–80)	0.173
Heart rate (/min)	80 (75–90)	89 (70–107)	0.022
SpO_2_ (%)	96 (95–96)	88 (86–96)	<0.001
Transplant duration (years)	6 (2–10)	10 (2–22)	0.157
Symptom duration upon hospital admission (days)	5 (3–10)	3 (1–8)	0.195
Acute kidney injury during follow-up	2 (3.8%)	6 (50.0%)	<0.001
Follow-up (days)	40 (37–43)	20 (14–35)	<0.001
**Comorbidities**			
Arterial hypertension	45 (84.9%)	8 (66.7%)	0.141
Diabetes mellitus	13 (24.5%)	5 (41.7%)	0.231
Chronic heart failure	2 (3.8%)	3 (25.0%)	0.013
Active smoking	5 (9.4%)	0 (0.0%)	0.268
Atrial fibrillation	6 (11.3%)	1 (8.3%)	0.763
Prior AMI	6 (11.3%)	1 (8.3%)	0.763
Prior CVI	4 (7.5%)	2 (16.7%)	0.324
PAD	7 (13.2%)	3 (25.0%)	0.307
COPD/asthma	4 (7.5%)	0 (0.0%)	0.326
**Laboratory parameters**			
WBC (×10^9^/L)	6.6 (5.2–8.1)	7.9 (3.9–9.9)	0.279
RBC (×10^12^/L)	4.7 (4.3–5.0)	4.3 (3.8–4.7)	0.089
Hgb (g/L)	133.0 (124.0–151.1)	127.5 (109.3–139.0)	0.059
Platelets (×10^3^/L)	213.0 (175.0–251.0)	204.5 (168.8–246.3)	0.618
CRP (mmol/L; maximal values)	72.6 (22.2–75.5)	102.8 (34.0–265.8)	0.003
BUN (mmol/L)	8.6 (6.6–12.7)	9.9 (6.0–17.0)	0.076
Creatinine (μmol/L)	134.5 (108.0–178.0)	147.0 (103.5–228.8)	0.187
eGFR (mL/min/1.73 m^2^)	42.5 (32.8–66.7)	37.5 (26.2–59.4)	0.264
D-dimers (mmol/L)	0.8 (0.5–1.1)	0.8 (0.7–1.5)	0.474

Data are expressed as number (percent) or median (interquartile range). **Abbreviations**: AMI—acute myocardial infarction; BUN—blood urea nitrogen; COPD—chronic obstructive pulmonary disease; CRP—C-reactive peptide; CVI—cerebrovascular incident; eGFR—estimated glomerular filtration rate; Hgb—haemoglobin; LDH—lactate dehydrogenase; PAD—peripheral arterial disease; RBC—red blood cells; WBC—white blood cells.

**Table 3:** Association and discrimination of different risk scores with 30-day post-discharge mortality (univariate Cox proportional hazards logistic regression analysis).

Risk Scores	30-Day Post-Discharge Mortality			
	HR (95% CI)	*p*-Value *	Harrell’s C Concordance Index	Somers’ D
**MEWS risk score**	1.65 (1.21–2.25)	0.002	0.694	0.388
**qCSI risk score**	1.32 (1.15–1.52)	<0.001	0.809	0.618
**VACO risk score**	1.00 (0.94–1.06)	0.904	0.427	−0.146
**PSI/PORT risk score**	1.04 (1.02–1.07)	0.001	0.833	0.665
**CCI risk score**	1.79 (1.13–2.83)	0.013	0.785	0.570
**MuLBSTA risk score**	1.31 (1.05–1.64)	0.017	0.678	0.356
**ISTH-DIC risk score**	1.72 (0.94–3.13)	0.077	0.645	0.289
**COVID-GRAM risk score**	1.03 (1.01–1.06)	0.004	0.748	0.496
**4C Mortality risk score**	1.79 (1.40–2.31)	<0.001	0.914	0.829

* Cox proportional hazards regression analysis (univariate model). **Abbreviations**: 4C Mortality Score—Coronavirus Clinical Characterisation Consortium Mortality Score; CCI—Charlson Comorbidity Index; COVID-GRAM—COVID-19 Critical Illness Prediction; ISTH-DIC—The International Society on Thrombosis and Haemostasis-Disseminated Intravascular Coagulation; MEWS—The Modified Early Warning Score; MuLBSTA—Multilobular infiltration, hypo-Lymphocytosis, Bacterial coinfection, Smoking history, hyper-Tension and Age; HR—hazard ratio; PSI/PORT—The Pneumonia Severity Index; qCSI—Quick COVID-19 Severity Index; VACO—Veterans Health Administration COVID-19 Index.

**Table 4:** Association of different risk scores with 30-day post-discharge mortality (multivariable Cox proportional hazards logistic regression analysis).

Risk Scores	30-Day Post-Discharge Mortality	
	aHR (95% CI)	*p*-Value *
**MEWS risk score**	1.56 (0.98–2.49)	0.059
**qCSI risk score**	1.33 (1.11–1.59)	0.002
**VACO risk score**	1.00 (0.99–1.01)	0.542
**PSI/PORT risk score**	1.04 (1.01–1.07)	0.012
**CCI risk score**	1.64 (0.94–2.84)	0.080
**MuLBSTA risk score**	1.36 (1.01–1.85)	0.046
**ISTH-DIC risk score**	1.40 (0.67–2.94)	0.376
**COVID-GRAM risk score**	1.03 (1.00–1.07)	0.095
**4C Mortality risk score**	1.93 (1.45–2.57)	<0.001

* Cox proportional hazards regression analysis (multivariable model). The model was adjusted for the following variables: MEWS (SpO_2_, CRP, chronic heart failure); qCSI (HR, SBP, CRP, chronic heart failure); VACO (HR, SBP, SpO_2_, CRP); PSI/PORT (CRP); CCI (HR, SBP, SpO_2_, CRP); MuLBSTA (HR, SBP, SpO_2_, CRP, chronic heart failure); ISTH-DIC (HR, SBP, SpO_2_, CRP, chronic heart failure); COVID-GRAM (HR, SBP, SpO_2_, CRP); 4C (HR, SBP). **Abbreviations**: aHR—adjusted hazard ratios; 4C Mortality Score—Coronavirus Clinical Characterisation Consortium Mortality Score; CCI—Charlson Comorbidity Index; COVID-GRAM—COVID-19 Critical Illness Prediction; ISTH-DIC—The International Society on Thrombosis and Haemostasis-Disseminated Intravascular Coagulation; MEWS—The Modified Early Warning Score; MuLBSTA—Multilobular infiltration, hypo-Lymphocytosis, Bacterial coinfection, Smoking history, hyper-Tension and Age; PSI/PORT—The Pneumonia Severity Index; qCSI—Quick COVID-19 Severity Index; VACO—Veterans Health Administration COVID-19 Index.

**Table 5:** ROC curve analysis of the risk scores with 30-day post-discharge mortality.

Risk Scores	C-Statistic (95% CI)	*p*-Value	Sensitivity/Specificity	Youden’s Index
**MEWS risk score**	0.705 (0.519–0.892)	0.027	50.0/90.6	>1
**qCSI risk score**	0.807 (0.650–0.965)	0.001	75.0/83.0	>2
**VACO risk score**	0.505 (0.340–0.670)	0.960	100.0/15.1	>3.6
**PSI/PORT risk score**	0.822 (0.714–0.929)	0.001	100.0/62.3	>82
**CCI risk score**	0.696 (0.534–0.857)	0.035	66.7/67.9	>5
**MuLBSTA risk score**	0.677 (0.527–0.827)	0.057	75.0/64.2	>10
**ISTH-DIC risk score**	0.611 (0.432–0.790)	0.233	83.3/34.0	>1
**COVID-GRAM risk score**	0.756 (0.622–0.889)	0.006	100.0/49.1	>125
**4C Mortality risk score**	0.854 (0.728–0.979)	<0.001	83.3/81.1	>10

**Abbreviations**: 4C Mortality Score—Coronavirus Clinical Characterisation Consortium Mortality Score; CCI—Charlson Comorbidity Index; COVID-GRAM—COVID-19 Critical Illness Prediction; ISTH-DIC—The International Society on Thrombosis and Haemostasis-Disseminated Intravascular Coagulation; MEWS—The Modified Early Warning Score; MuLBSTA—Multilobular infiltration, hypo-Lymphocytosis, Bacterial coinfection, Smoking history, hyper-Tension and Age; OR—odds ratio; PSI/PORT—The Pneumonia Severity Index; qCSI—Quick COVID-19 Severity Index; VACO—Veterans Health Administration COVID-19 Index.

## Data Availability

The datasets used and/or analysed during the current study are available from the corresponding author upon reasonable request.

## Supplementary Materials

The following supporting information can be downloaded at: https://www.mdpi.com/article/10.3390/medicina59040657/s1. It includes Supplementary Material: Supplementary Figure S1. Flow diagram of the study; Supplementary Table S1. Elementary data about selected risk scores from the study; Supplementary Table S2. Other characteristics and risk scores of the study sample; Supplementary Table S3. Calibration of different risk scores with 30-day post-discharge mortality; Supplementary Table S4. Comparison of ROC curves between selected risk scores.

## Appendix A

**The Strengthening the Reporting of Observational Studies in Epidemiology (STROBE) Checklist—includes a point-by-point report of this study in accordance to the guidelines for reporting observational studies**.
