# Supplementary material for: Association of Different Risk Scores and 30-Day Mortality in Kidney Transplant Recipients with COVID-19
Source: Medicina (Kaunas). 2023 Mar 26;59(4):657. doi: 10.3390/medicina59040657 (PMC10146380; doi:10.3390/medicina59040657)

SUPPLEMENTARY TABLES

Supplementary Table S1. Elementary data about selected risk scores from the

Risk scores	Important features		
	**Year of derivation**	**Study population**	**Score range**
**MEWS risk score**	2001	General patients admitted to the emergency department	0-14
**qCSI risk score**	2020	Hospitalized COVID-19 patients	0-12
**VACO risk score**	2020	Patients who tested positive for SARS-CoV-2 in inpatient or outpatient settings	/
**PSI/PORT risk score**	1997	Patients with CAP	1-5 risk groups
**CCI risk score**	1987	General hospitalized patients	0-37
**MuLBSTA risk score**	2019	Patients with viral pneumonia	0-20
**ISTH-DIC risk score**	2001	General hospitalized patients	0-8
**COVID-GRAM risk score**	2020	Hospitalized COVID-19 patients	1-3 risk groups
**4C Mortality Score**	2020	Hospitalized COVID-19 patients	0-20

Abbreviations: 4C Mortality Score– Coronavirus Clinical Characterisation Consortium Mortality Score; CCI – Charlson Comorbidity Index; COVID-GRAM – COVID-19 Critical Illness Prediction; ISTH-DIC – The International Society on Thrombosis and Haemostasis-Disseminated Intravascular Coagulation; MEWS – The Modified Early Warning Score; MuLBSTA – Multilobular infiltration, hypo-Lymphocytosis, Bacterial coinfection, Smoking history, hyper-Tension and Age; OR – odds ratio; PSI/PORT – The Pneumonia Severity Index; qCSI – Quick COVID-19 Severity Index; VACO – Veterans Health Administration COVID-19 Index, CAP – community-acquired pneumonia, SARS CoV-2 – Sever acute respiratory syndrome coronavirus 2.

Supplementary Table S2. Other characteristics and risk scores of the study sample.

Variables	Study sample (N=65)
**Subfebrility**	28 (43.1%)
**Febrility**	29 (44.6%)
**Cough**	36 (55.4%)
**Dyspnea**	15 (23.1%)
**Headache**	16 (24.6%)
**Sore throat**	13 (20.0%)
**Malaise**	49 (75.4%)
**Rhinorrhea**	3 (4.6%)
**Anosmya**	4 (6.2%)
**Diarrhea**	13 (20.0%)
**Venous thromboembolism**	1 (1.5%)
**X-ray confirmed pneumonia**	46 (70.8%)
**X-ray confirmed pleural effusion**	2 (3.1%)
**Positive urine culture**	9 (13.8%)
**Positive blood culture**	4 (6.2%)
**Risk scores**	
**MEWS risk score**	1 (0, 1)
**qCSI risk score**	1 (0, 4)
**VACO risk score**	12 (6, 18)
**PSI/PORT risk score**	82 (67, 101)
**CCI risk score**	5 (3, 6)
**MuLBSTA risk score**	9 (7, 13)
**ISTH-DIC risk score**	2 (0, 2)
**COVID-GRAM risk score**	130 (112, 142)
**4C Mortality Score**	8 (6, 11)

Data are expressed as number (percent) or median (interquartile range).
Abbreviations: None.

Supplementary Table S3. Calibration of different risk scores with 30-day post-discharge mortality.

Risk scores	***30-day post-discharge mortality***	
	**Hosmer-Lemeshow test**	**p-value**
**MEWS risk score**	1.57	0.211
**qCSI risk score**	0.51	0.775
**VACO risk score**	8.89	0.351
**PSI/PORT risk score**	9.16	0.329
**CCI risk score**	1.75	0.781
**MuLBSTA risk score**	4.33	0.364
**ISTH-DIC risk score**	1.89	0.389
**COVID-GRAM risk score**	15.71	0.047
**4C risk score**	7.61	0.268

Abbreviations: 4C – 4C Mortality; CCI – Charlson Comorbidity Index; COVID-GRAM – COVID-19 Critical Illness Prediction; ISTH-DIC – The International Society on Thrombosis and Haemostasis-Disseminated Intravascular Coagulation; MEWS – The Modified Early Warning Score; MuLBSTA – Multilobular infiltration, hypo-Lymphocytosis, Bacterial coinfection, Smoking history, hyper-Tension and Age; OR – odds ratio; PSI/PORT – The Pneumonia Severity Index; qCSI – Quick COVID-19 Severity Index; VACO – Veterans Health Administration COVID-19 Index.

Supplementary Table S4. Comparison of ROC curves between selected risk scores*

Risk scores	MEWS	qCSI	PSI/PORT	CCI	COVID-GRAM	4C
**MEWS**		0.102<br>p=0.365	0.116<br>p=0.265	0.009<br>p=0.931	0.050<br>p=0.665	0.149<br>p=0.181
**qCSI**			0.014<br>p=0.842	0.112<br>p=0.224	0.052<br>p=0.503	0.046<br>p=0.576
**PSI/PORT**				0.126<br>p=0.036	0.067<br>p=0.235	0.032<br>p=0.527
**CCI**					0.060<br>p=0.433	0.158<br>p=0.010
**COVID-GRAM**						0.098<br>p=0.078
**4C**						

* Data are presented as the difference between areas - method by *Hanley & McNeil* (1983).

Abbreviations: 4C Mortality Score – Coronavirus Clinical Characterisation Consortium Mortality Score; CCI – Charlson Comorbidity Index; COVID-GRAM – COVID-19 Critical Illness Prediction; ISTH-DIC – The International Society on Thrombosis and Haemostasis-Disseminated Intravascular Coagulation; MEWS – The Modified Early Warning Score; MuLBSTA – Multilobular infiltration, hypo-Lymphocytosis, Bacterial coinfection, Smoking history, hyper-Tension and Age; OR – odds ratio; PSI/PORT – The Pneumonia Severity Index; qCSI – Quick COVID-19 Severity Index; ROC – Receiver Operator Characteristics.

Supplementary Figure S1: Flow Diagram

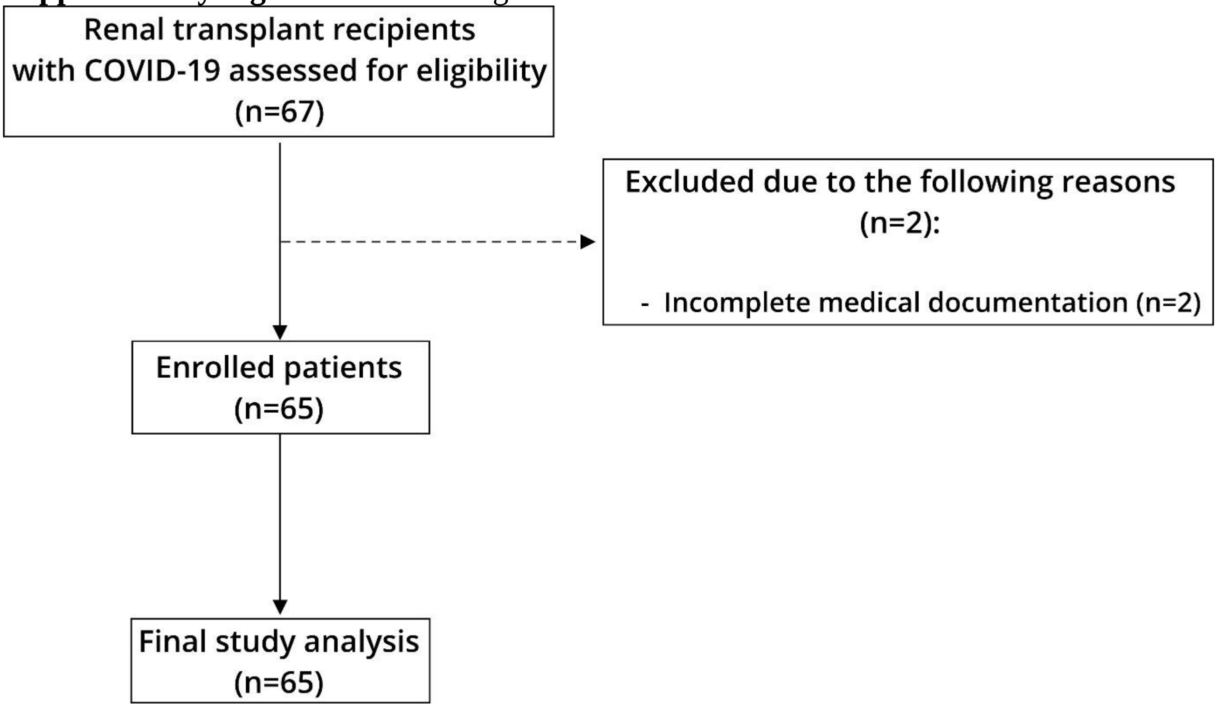

Supplement: Supplementary file 1 [file medicina-59-00657-s001.zip › medicina-2269024-supplementary.pdf]
